# Characterization of pulmonary embolism events in patients with suspected pneumonia

**DOI:** 10.1007/s11739-026-04496-7

**Published:** 2026-08-18

**Authors:** Ariel Kenig, Tal Sigawi, Josef Daniel Shakargy, Fares Darawshy, Rottem Kuint, Henny Azmanov, Neville Berkman

**Affiliations:** 1 Department of Medicine, Hadassah Medical Center, Faculty of Medicine, Hebrew University, Jerusalem, Israel; 2 Institute of Pulmonary Medicine, Hadassah Medical Center, Faculty of Medicine, Hebrew University, Jerusalem, Israel

**Keywords:** Pulmonary embolism, Pneumonia, D-dimer, CT pulmonary angiography

## Abstract

Pneumonia and pulmonary embolism (PE) share similar clinical presentations, posing a significant diagnostic challenge. We investigated whether available diagnostic modalities could identify distinct characteristics in patients suspected of pneumonia later diagnosed with PE. Patients who underwent a CT pulmonary angiography (CTPA) scan on admission were retrospectively screened for suspected pneumonia, defined as a chest X-ray infiltrate accompanied by clinical or laboratory evidence of infection. Patients with COVID-19, pregnancy, and thrombophilia were excluded. Comorbidities, clinical presentation, electrocardiograms, laboratory tests, and outcomes were compared between patients with and without PE. Among 5,522 CTPA scans, 846 patients had suspected pneumonia. 106 (12.5%) had PE. Patients with PE had a higher rate of prior venous thromboembolism (14.2% vs. 7.7%, *P* = 0.03). However, other risk factors and baseline anticoagulation therapy were comparable between groups. Most clinical and laboratory parameters, including CRP and D-dimer levels, were similar, as were the Wells criteria and Geneva scores. New-onset atrial fibrillation was more frequent in PE patients (11.1% vs. 5.4%, *P* = 0.009). Prolonged expiration and wheezing were more common in those without PE (17.9% vs. 5.7%, *P* = 0.0015) and remained the only discriminative parameter in a multivariate analysis (OR = 0.29, 95%CI: 0.09–0.74, *P* = 0.021). Notably, the presence of PE did not impact mortality rates. PE is prevalent in patients with suspected pneumonia undergoing CTPA. Clinical presentation, laboratory findings, and commonly used diagnostic tools, including the Wells criteria and the Geneva score, perform poorly in this setting. These findings underscore the need for new diagnostic approaches.

**Supplementary Information:**

The online version contains supplementary material available at https://doi.org/10.1007/s11739-026-04496-7.

## Introduction

Pulmonary embolism (PE) is a significant cause of morbidity and mortּality [1]. The diagnosis of PE might be missed or delayed in patients with suspected pneumonia due to overlapping clinical and laboratory manifestations.[2, 3]. The possible co-occurrence of PE and pneumonia further complicates the diagnosis and should be considered in cases of non-resolving pneumonia [2, 4–7].

Symptoms of pneumonia, such as dyspnea and cough, are frequently seen in patients with PE [1, 7]. Furthermore, patients with PE occasionally present with fever and elevated inflammatory markers [8]. D-dimer is a widely used and sensitive marker for acute thromboembolic events and is pivotal in stratifying PE probability and guiding the decision to pursue computed tomography pulmonary angiography (CTPA) [1, 9, 10]. However, D-dimer is also a non-specific acute-phase reactant that can be elevated in inflammatory and infectious states, including pneumonia [1, 9, 10]. The use of age-adjusted D-dimer and cut-offs adapted to clinical probability, as recommended by recent guidelines, improves its utility to some extent [1, 11, 12]. Chest X-ray (CXR), a common diagnostic tool for pneumonia, lacks sensitive and specific findings for PE[13]. Moreover, a pulmonary infarct on CXR may be misinterpreted as an infectious infiltrate [14, 15]. These factors complicate the differentiation between PE and pneumonia.

Effective predictive tools to identify acute PE in patients with suspected pneumonia are crucial to prevent misdiagnosis, which can result in inadequate treatment, increased mortality, and increased risk of long-term sequelae of PE, such as chronic thromboembolic pulmonary hypertension (CTEPH) [1, 16, 17]. Furthermore, these tools could help minimize adverse events and costs associated with unnecessary CTPA; however, their utility varies by patient characteristics and clinical setting [18, 19]. Whether the currently available tools, including history-taking, physical examination, laboratory evaluation, ECG, and PE prediction scores, are sufficient for this purpose has not been established. Defining this diagnostic gap could facilitate the adoption of novel PE diagnostic modalities, including new prediction models, point-of-care ultrasound (POCUS), and artificial intelligence (AI) [20–26].

Although PE and pneumonia are among the most common conditions encountered by physicians, the implications of PE diagnosis in those with suspected pneumonia have not been thoroughly studied [2, 3]. In this study, we aimed to identify the characteristics of PE in patients presenting to the emergency department with suspected community-acquired pneumonia and to evaluate the utility of available diagnostic modalities in identifying these patients.

## Methods

### Study design

We conducted a single-center, retrospective study at Hadassah Medical Center, Jerusalem, Israel. The study was approved by the Institutional Ethics Review Board (HMO-0080–23). We reviewed electronic medical records of all patients aged 18 years or older who underwent CTPA between January 2021 and December 2022. We initially excluded patients who lacked a CXR before CTPA, those in whom CTPA was performed more than 3 days after hospitalization (to avoid including hospitalization-provoked PE), and scans deemed technically inadequate by the radiologist's report. Patients with active COVID-19, a major cause of thromboembolism, were also excluded to enhance the generalizability of the findings to non-COVID-19 CAP. Finally, patients with known thrombophilia or those who were pregnant were also excluded.

Cases meeting the inclusion and exclusion criteria were screened for suspected pneumonia. Suspected pneumonia was defined as the presence of a CXR infiltrate accompanied by at least one of the following: reported cough or sputum production, reported or measured temperature ≥ 38 °C, C-reactive protein (CRP) level ≥ 2 mg/dL, or white blood cell count ≥ 11 × 10^9^ cells/L. CXRs were independently evaluated by two qualified physicians. Inclusion required agreement on the presence of pulmonary infiltrates. Cases were classified according to the presence or absence of PE based on CTPA findings. Patients who did not meet the radiological or clinical definition of suspected pneumonia were excluded from the analysis.

### Data collection

Patient data were collected, including the history of present illness, findings on physical examination, comorbidities, presence of risk factors for venous thromboembolism (VTE; including prior VTE, surgery, or fracture within 30 days of admission, or immobilization), laboratory results, the Wells and Geneva scores, electrocardiographic (ECG) tracings, CT reports, and clinical outcomes. The clinical outcomes assessed included hospitalization duration, 30-day, 90-day, and 1-year mortality rates, transfer to the intensive care unit (ICU), and the need for mechanical ventilation and vasopressor support.

### Statistical analysis

Descriptive statistics were used to summarize baseline characteristics, with continuous variables presented as means and standard deviations or medians and interquartile ranges, as appropriate, and categorical variables as frequencies and percentages. Continuous variables were compared using the t test or the Mann–Whitney U test, as appropriate. Categorical variables were analyzed using the Chi-square test or Fisher’s exact test. A *p* value of < 0.05 was considered statistically significant. The p values reported in Table 1 are descriptive and were therefore not adjusted for multiple comparisons. However, an analysis applying the Bonferroni correction was also performed, and is addressed in the discussion.

**Table 1:** Baseline characteristics

Characteristics	No. (%)^1^		*p* value
	PE-negative *N* = 740	PE-positive *N* = 106	
Gender			0.13
Female	344 (46.5)	41 (38.7)	
Male	396 (53.5)	65 (61.3)	
Age, years, mean (SD)	69 (16)	69 (17)	0.74
Diabetes mellitus	259 (35.0)	27 (25.5)	0.052
Hypertension	420 (56.8)	49 (46.2)	**0.041**
Ischemic heart disease	157 (21.2)	19 (17.9)	0.44
Atrial fibrillation	107 (14.5)	7 (6.6)	**0.027**
Heart failure	206 (27.8)	15 (14.2)	**0.003**
Cerebrovascular accident	89 (12.0)	11 (10.4)	0.62
Obesity	143 (19.4)	21 (19.8)	0.91
Solid malignancy	195 (26.4)	29 (27.4)	0.83
Hematologic malignancy	32 (4.3)	3 (2.8)	0.61
Chronic lung disease	266 (35.9)	37 (34.9)	0.84
Chronic liver disease	13 (1.8)	1 (0.9)	1.00
Chronic kidney disease	72 (9.7)	5 (4.7)	0.09
Dialysis	21 (2.8)	4 (3.8)	0.54
Inflammatory disease	42 (5.7)	4 (3.8)	0.42
Smoking status			0.89
Current	146 (19.7)	23 (21.7)	
Never	509 (68.8)	71 (67.0)	
Past	85 (11.5)	12 (11.3)	
Previous VTE	57 (7.7)	15 (14.2)	**0.026**
Recent surgery^2^	30 (4.1)	4 (3.8)	1.00
Immobilization	173 (23.4)	34 (32.1)	0.051
Recent fracture ^2^	25 (3.4)	4 (3.8)	0.78
Therapeutic anticoagulation	125 (16.9)	12 (11.3)	0.15
Preventive anticoagulation	28 (3.8)	6 (5.7)	0.42
Antiplatelet	237 (32.1)	25 (23.4)	0.07
Central venous catheter	60 (8.1)	15 (14.2)	**0.041**
Fever or chills	194 (26.2)	29 (27.4)	0.80
Cough	318 (43.0)	48 (45.3)	0.65
Phlegm	183 (24.8)	20 (18.7)	0.17
Hemoptysis	28 (3.8)	4 (3.8)	1.00
Chest pain	192 (25.9)	29 (27.4)	0.76
Shortness of breath	556 (75.1)	72 (67.9)	0.11
Loss of consciousness	40 (5.4)	10 (9.4)	1.00
Unilateral leg pain	9 (1.2)	1 (0.9)	1.00
Bilateral leg edema	175 (23.7)	29 (27.4)	0.41
Signs of DVT	29 (3.9)	10 (9.4)	**0.022**
Focal auscultation abnormalities ^3^	465 (62.9)	72 (67.9)	0.32
Wheezing or prolonged expiration ^4^	132 (17.9)	6 (5.7)	**0.0015**
	Mean (± SD)^1^		
Temperature^5^, °C	37.05 (0.88)	37.14 (0.93)	0.83
Systolic blood pressure, mmHg	131 (28)	119 (26)	**0.0002**
Diastolic blood pressure, mmHg	73 (16)	70 (15)	0.26
Heart rate, bpm	100 (22)	98 (25)	0.35
Respiratory rate,	28 (8)	25 (8)	**0.039**
Oxygen saturation,	88 (10)	89 (8)	0.43
Oxygen supplementation^6^, No. (%)	443 (66.7)	57 (58.2)	0.096
Creatinine, mg/dL	99.1 (91.5)	108.8 (114.3)	0.13
ALT, U/L	53.7 (440.5)	42.2 (87.2)	0.14
ALP, U/L	131.6 (122.2)	131.5 (136.4)	0.47
Total bilirubin, µmol/L	12.2 (13.7)	16.4 (25.6)	**0.011**
PCO_2_^7^	46.2 (14.1)	43.1 (12.2)	**0.040**
Hemoglobin, g/dL	12.1 (2.3)	12.4 (2.6)	0.25
White blood cells, × 10^9^ cells/L	11.9 (6.7)	11.7 (8.1)	0.61
Platelets, × 10^9^ cells/L	293.5 (153.0)	249.9 (135.6)	**0.001**
ESR, mm	44.8 (21.4)	48.5 (14.0)	0.67
CRP, mg/dL	9.6 (9)	11.3 (8)	**0.002**
INR	1.1 (0.3)	1.1 (0.1)	**0.046**
PTT, sec	29.4 (6.4)	30.0 (6.3)	0.43
Troponin ng/L	187.6 (849.2)	136.9 (405.3)	0.35
D-dimer, mg/dL	5.1 (8.7)	8.9 (16.0)	**0.002**
Pro-BNP, pg/mL	4,942 (7,853)	7,824 (15,737)	0.091
Lactate, mmol/L	2.6 (1.8)	2.6 (2.2)	0.75

*ALT* alanine aminotransferase, *ALP* alkaline phosphatase, *BNP* brain natriuretic peptide, *CRP* C-reactive protein, *ESR* erythrocyte sedimentation rate, *INR* international normalization ratio, *PE* pulmonary embolism, *PTT* partial thromboplastin time, *RDW* red blood cell distribution width, *VTE* venous thromboembolism

^1^Unless stated otherwise

^2^In the past 30 days

^3^Including decreased breath sounds, bronchial breathing, rales, and rhonchi

^4^According to the examining physician's documentation in the electronic medical record

^5^Body temperature according to a single measurement recorded from the emergency department admission

^6^The types of oxygen supplementation in patients with and without PE included: nasal cannula (41.8% and 40.6%), face mask (11.2% and 20.0%), high flow nasal cannula (1.0% and 0.3%), non-invasive ventilation (1.0% and 0.9%), and mechanical ventilation (5.0% and 3.0%). The distribution of supplementation type was similar between groups (*P* = 0.18)

^7^PCO_2_ values were obtained from venous blood gas analyses. The distribution was not significantly different (*P* =)

we have used bold for p < 0.05

Our primary objective was to identify predictive characteristics independently associated with PE among patients with suspected pneumonia. Therefore, variables showing both statistically significant (*p* < 0.05) and clinically relevant differences between groups were included, along with age and sex, in the univariate and multivariate analyses. All selected variables were entered into the model simultaneously, without stepwise selection. Multicollinearity was assessed using the variance inflation factor (VIF), and all parameters had VIF < 2, indicating a low risk of collinearity. A post hoc sensitivity analysis was conducted using a reduced multivariable logistic regression model based on the main model, retaining only variables from the original model that are directly relevant to the routine clinical evaluation of pulmonary embolism.

The diagnostic performance of the Geneva score, the Wells criteria, and positive D-dimer was calculated in the study population. Positive Wells criteria were defined as a score ≥ 2 or a score of 0–1 combined with D-dimer levels > 0.5 mg/dL. A positive Geneva score was a score of 4–10 with a positive D-dimer or a score > 10 regardless of D-dimer levels. To assess the performance of D-dimer, CRP, and troponin in patients with suspected pneumonia evaluated for PE, their distribution and receiver-operating characteristic area under the curve (ROC-AUC) were computed. All statistical analyses were performed with R software (version 4.3.2).

## Results

### Baseline patient characteristics

**Fig. 1:**
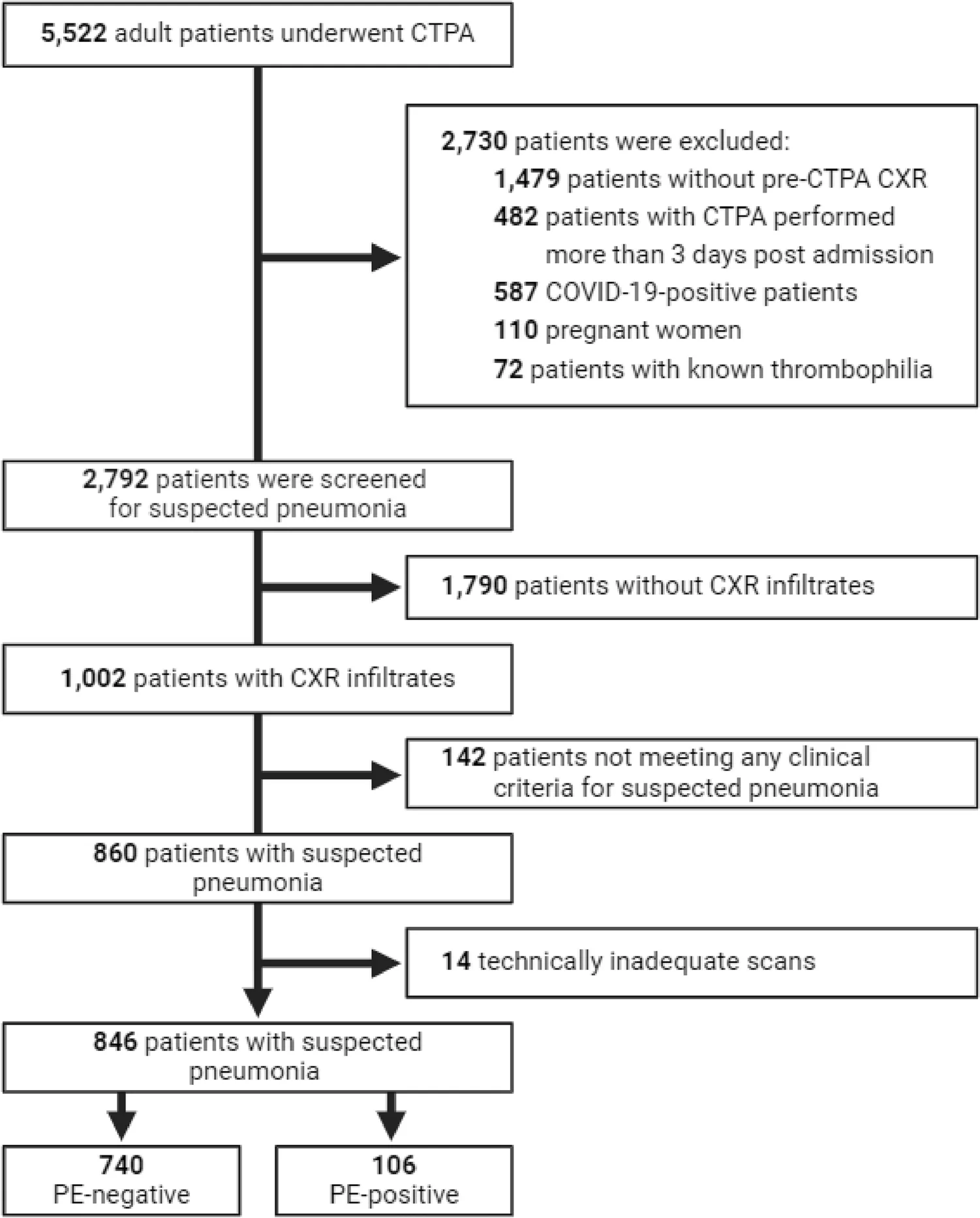
Study design. 5,522 patients who underwent CTPA were screened for eligibility according to inclusion and exclusion criteria. Patients without CXR, with CTPA performed later than 3 days from admission, those positive for COVID-19, pregnant women, or patients with known thrombophilia were excluded. Thereafter, cases were assessed for radiological and clinical criteria for suspected pneumonia. Finally, 846 patients were included; among them, 106 had PE. *COVID-19* coronavirus disease, *CTPA* computed tomography pulmonary angiography, *CXR* chest X-ray, *PE* pulmonary embolism

A total of 5,522 CTPA cases were screened. After excluding 4,676 cases, 846 cases of suspected pneumonia were included in the final analysis (Fig. 1). Of these, 106 (12.5%) were eventually diagnosed with PE. Central pulmonary arteries were involved in 30 (28.3%) patients, lobar arteries in 16 (15.1%) patients, segmental arteries in 40 (37.7%) patients, and subsegmental arteries in 20 (18.9%) patients.

The baseline demographic features, underlying medical conditions, clinical presentation, and laboratory findings of patients with and without PE are detailed in Table 1. The mean age was 69 in both groups, with a slight male predominance (61.3% and 53.5% in the PE-positive and PE-negative groups, respectively, *P* = 0.13). Patients in the PE-positive group had a significantly higher rate of prior VTE than PE-negative patients (14.2% vs. 7.7%, *P* = 0.026). However, other risk factors for VTE, such as the presence of malignancy, recent surgery, or leg fracture, were similar between groups. PE-negative patients had significantly higher rates of preexisting hypertension (56.8% vs. 46.2%, *P* = 0.041), atrial fibrillation (AF; 14.5% vs. 6.6%, *P* = 0.027), and heart failure (27.8% vs. 14.2%, *P* = 0.003).

### Clinical presentation

Overall, the clinical presentation was similar among patients with suspected pneumonia, regardless of PE presence. Signs of deep vein thrombosis (DVT) were more common in PE-positive patients (9.4% vs. 3.9%, *P* = 0.022). In contrast, PE-negative patients had a higher prevalence of signs compatible with airflow obstruction, including wheezing or prolonged expiration (17.9% vs. 5.7%, *P* = 0.0015). PE-negative patients also had slightly higher systolic blood pressure (131 ± 28 vs. 119 ± 26 mmHg, *P* < 0.001) and respiratory rate (28 ± 8 vs. 25 ± 8 breaths per minute, *P* = 0.039).

### Laboratory and ECG findings

**Fig. 2:**
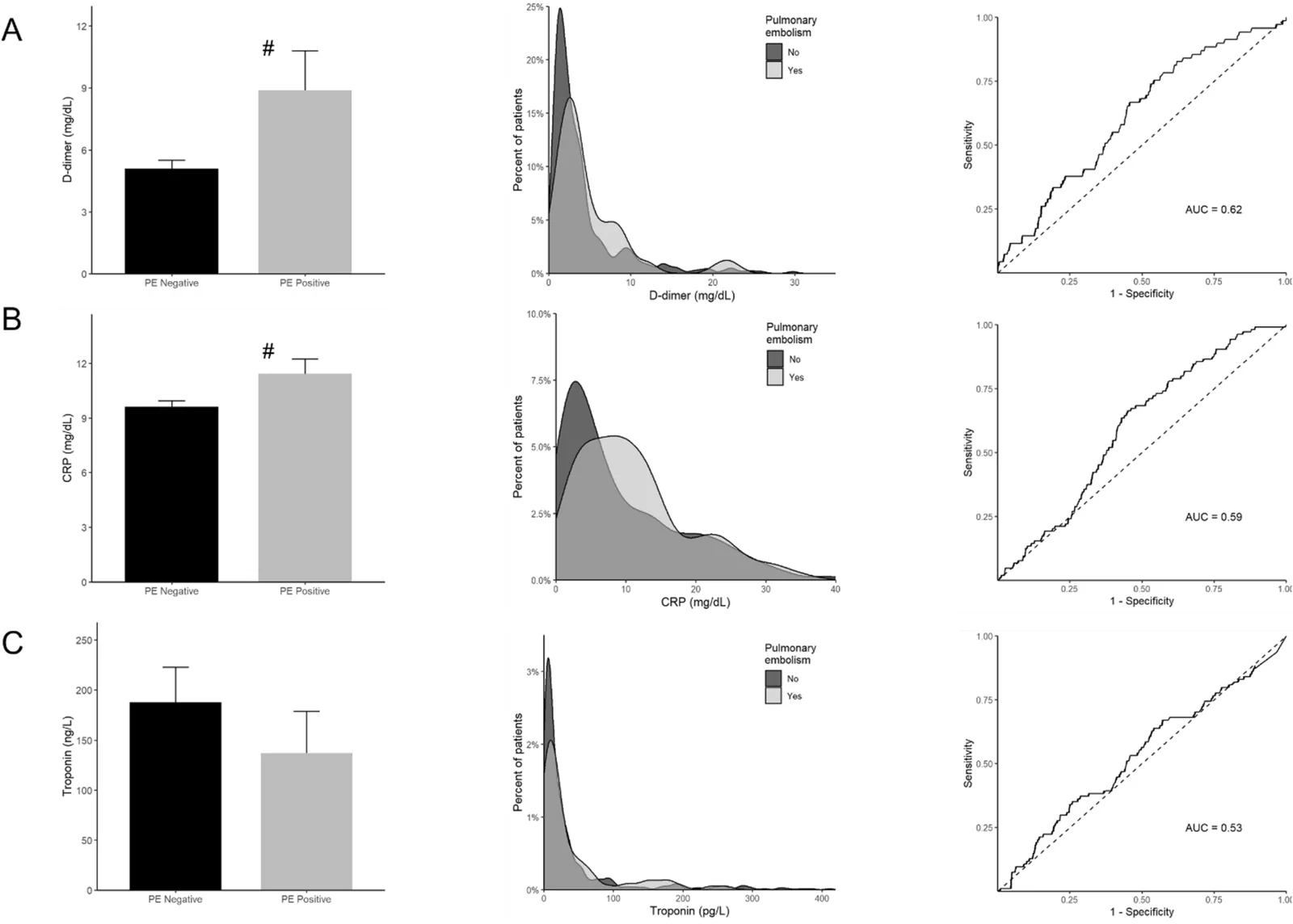
Levels, distribution, and ROC curves for D-Dimer (**A**), CRP (**B**), and troponin (**C**) in patients with suspected pneumonia with and without PE. *AUC* area under the curve, *CRP* C-reactive protein, *PE* pulmonary embolism, *ROC* receiver-operator curves

Laboratory tests revealed mean D-dimer levels above the upper limit of normal in both groups, with higher levels observed in the PE-positive group (8.9 ± 16 vs. 5.1 ± 8.7 mg/dL, *P* = 0.002). CRP was also increased in both groups, with significantly higher levels in the PE-positive group (11.3 ± 8 vs. 9.6 ± 9 mg/dL, *P* = 0.002). Other laboratory findings are summarized in Table 1. To assess whether specific laboratory markers could predict PE diagnosis among patients with suspected pneumonia, we evaluated the distributions and ROC-AUC for CRP, troponin, and D-dimer (Fig. 2). All markers demonstrated poor discriminative efficacy between PE-positive and PE-negative suspected pneumonia patients, with ROC-AUC values ranging from 0.53 to 0.62.

The prevalence of sinus tachycardia, right axis deviation, signs of right-ventricular strain, S1Q3T3 pattern, and additional ECG findings was similar in both groups (Online resources, Table S1). However, new-onset AF, defined as an ECG finding consistent with AF in a patient without a prior diagnosis, was more common among PE-positive patients (11.1% vs. 5.4%, *P* = 0.009).

### Outcomes

Patients with suspected pneumonia who were subsequently found to have PE were more frequently hospitalized (91.5% vs. 82%, *P* = 0.015). No between-group differences were noted in 30-day, 90-day, and 1-year mortality rates, ICU admissions, length of hospitalization, or the need for mechanical ventilation or vasopressor support (Table 2 and Figure S1**,** in the online resources). A subanalysis excluding subsegmental PE also failed to show a difference in outcomes between groups (Online resources, Table S3).

**Table 2:** Outcomes

	No. (%)		
Characteristic	PE-negative *N* = 740	PE-positive *N* = 106	*p* value
Mortality			
Total	331 (44.7)	47 (44.3)	0.94
30-day mortality	87 (11.8)	8 (7.5)	0.20
90-day mortality	133 (18.0)	14 (13.2)	0.23
One-year mortality	179 (24.2)	24 (22.6)	0.73
Hospitalization	607 (82.0)	97 (91.5)	**0.015**
Length of stay, days	5 (2–9)	5 (3–11)	0.10
ICU admission	58 (7.8)	12 (11.3)	0.22
Mechanical ventilation	74 (10.0)	12 (11.3)	0.74
Vasopressor support	67 (9.1)	11 (10.4)	0.66

*ICU* intensive care unit

*we have used bold for p < 0.05*

### PE prediction tools

PE prediction tools were positive in most cases, as expected, since CTPA was a prerequisite for inclusion (Table 3). We assessed potential numerical differences in available PE prediction tools in the setting of suspected pneumonia. The Wells score was significantly higher in the PE-positive group than the PE-negative group, although the clinical importance of the difference is uncertain (2.05 ± 1.54 vs. 1.62 ± 1.31, *P* = 0.006). However, when incorporating Wells criteria and D-dimer values, both groups showed a high and similar proportion of positive scores (97.2% vs. 98.3%, *P* = 0.63). No statistically significant difference was observed in the Geneva score values or positivity rate (5.70 ± 2.70 vs. 5.55 ± 2.27, *P* = 0.48, and 71.6% vs. 76.4%, *P* = 0.37, PE-positive vs. PE-negative groups). The diagnostic performances of the Wells criteria, the Geneva score, and D-dimer values are presented in Table 4. Both the Wells and Geneva scores exhibited low specificity and negative predictive values. A subanalysis excluding subsegmental PE did not improve the predictive accuracy (Online resources, Table S4). The Wells criteria and the Geneva score showed poor diagnostic differentiation in our population, with an AUC of 0.52 for the Geneva score and 0.58 for the Wells criteria.

**Table 3:** Wells criteria and Geneva score in patients with suspected pneumonia. Comparison of Wells criteria and Geneva score in patients with and without PE

Characteristic	PE-negative *N* = 470	PE-positive *N* = 106	*p* value
Wells score, mean (SD)	1.62 (1.31)	2.05 (1.54)	**0.006**
Geneva score, mean (SD)	5.55 (2.27)	5.70 (2.70)	0.48
Positive Wells criteria^1^, n (%)	407 (98.3)	69 (97.2)	0.63
Positive Geneva score^2^, n (%)	337 (76.4)	53 (71.6)	0.37

*PE* pulmonary embolism

^1^Positive Wells criteria were defined as a score ≥ 2 or 0–1 combined with D-dimer levels > 0.5 mg/dL

^2^A positive Geneva score was defined as a score of 4–10 with a positive D-dimer or a score > 10 regardless of D-dimer levels

we have used bold for p < 0.05

**Table 4:** Wells criteria, Geneva score, and D-dimer performances in suspected pneumonia

Test	Sensitivity	Specificity	PPV	NPV
Wells criteria (%)	97.2	1.7	14.5	77.8
Geneva score (%)	71.6	23.6	13.6	83.2
D-dimer > 0.5 mg/dL (%)	97.1	2.0	13.2	81.8
D-dimer > 1 mg/dL (%)	94.2	13.3	14.3	93.8
Age-adjusted D-dimer^1^ (%)	95.7	5.8	13.4	89.7

*NPV* negative predictive value, *PPV* positive predictive value

^1^Age-adjusted D-dimer cutoff relates to the increase of the upper normal limit (0.5 mg/dL) by 0.1 mg/dL for every decade above 50 years of age

### Multivariate analysis

Variables demonstrating both statistically and clinically significant differences between the two study groups were included, along with age and sex, in the univariate and multivariate logistic regression analyses (Table 5). In the univariate analysis, new-onset AF was associated with increased odds of diagnosing PE (OR 3.75, 95% CI 1.64 to 8.08, *P* = 0.001). Conversely, the presence of wheezing or prolonged expiration on auscultation was strongly associated with a reduced likelihood of PE, and this remained the only statistically significant variable in the multivariate model (OR 0.29, 95%CI 0.09–0.74, *P* = 0.021) for PE diagnosis. A baseline diagnosis of heart failure, hypertension, or AF was linked to lower odds of PE diagnosis in the univariate analysis, although these associations did not persist in the multivariate model. A post hoc analysis was conducted (Online resources, Table S5), retaining from the main model age and variables routinely assessed in the evaluation of PE (previous VTE, signs of DVT, and D-dimer levels). Only D-dimer was independently associated with an increased risk of PE, although the effect size was small (OR 1.02, 95% CI 1.00–1.04, *P* = 0.015).

**Table 5:** Univariate and multivariate analyses of parameters associated with a diagnosis of pulmonary embolism in patients with suspected pneumonia

Characteristic	Univariate			Multivariate		
	OR	95% CI	*p* value	OR	95% CI	*p* value
Age	1.00	0.99 to 1.01	0.97	1.00	0.98 to 1.02	0.88
Gender, male	0.73	0.48 to 1.10	0.13	0.74	0.43 to 1.28	0.29
Hypertension	0.66	0.43 to 0.99	**0.04**	0.60	0.33 to 1.08	0.09
Known AF	0.42	0.17 to 0.86	**0.03**	0.30	0.05 to 1.08	0.12
New-onset AF^1^	3.75	1.64 to 8.08	**0.001**	2.26	0.75 to 6.03	0.12
Heart failure	0.43	0.23 to 0.73	**0.003**	0.60	0.27 to 1.25	0.19
Previous VTE	1.98	1.04 to 3.55	**0.03**	1.48	0.60 to 3.33	0.36
Central venous catheter	1.87	1.02 to 3.42	**0.04**	1.43	0.56 to 3.26	0.42
Signs of DVT	2.55	1.15 to 5.24	**0.01**	1.93	0.62 to 5.27	0.22
Wheezing or prolonged expiration	0.28	0.11 to 0.59	**0.003**	0.29	0.09 to 0.74	**0.02**
D-dimer, mg/dL	1.03	1.01 to 1.05	**0.007**	1.02	0.99 to 1.04	0.08

*AF* atrial fibrillation, *CI* confidence interval, *DVT* deep vein thrombosis, *OR* odds ratio, *PE* pulmonary embolism, *VIF* variance inflation factor, *VTE* venous thromboembolism

Characteristics that presented a statistically and clinically significant difference between PE-positive and PE-negative patients were included in the univariate analysis. All characteristics were subsequently tested in the multivariate analysis to identify independent predictors of PE among patients with suspected pneumonia. Multicollinearity was tested by calculating VIF. VIF values ranged from 1.01 to 1.26, indicating a low risk of multicollinearity

^1^New-onset atrial fibrillation was defined as AF on admission without documentation of AF in the patient’s medical history

we have used bold for p < 0.05

## Discussion

Differentiating pneumonia from PE is an essential yet often challenging task for the diagnosing physician, as both conditions share similar clinical presentations and risk factors [1, 5, 15, 27, 28]. Each condition may also increase the likelihood of the other occurring [6, 14, 29]. Therefore, clinicians may encounter variable clinical scenarios, as PE might follow, predispose, co-occur, or mimic pneumonia [4–6]. Since pneumonia is more common than PE, physicians might fall victim to familiarity bias, attributing patients' symptoms and laboratory findings to an infectious process rather than VTE [1, 28]. On the other hand, performing CTPA for every patient with suspected pneumonia in the emergency department is impractical and carries risks and costs. Therefore, reliable diagnostic tools are crucial for detecting PE in this common clinical scenario.

A substantial proportion of patients with suspected pneumonia were diagnosed with PE, with a detection rate of 12.5%. However, available diagnostic tests and PE decision tools underperform in these patients. Most signs, symptoms, VTE risk factors, laboratory tests, and ECG findings were similar or showed no clinically significant differences between patients with suspected pneumonia who had PE and those who did not, and therefore could not reliably differentiate suspected pneumonia patients with and without PE. Furthermore, applying the Bonferroni correction for multiple comparisons to the baseline characteristics in Table 1 results in most between-group differences losing significance. D-dimer was elevated in both groups, with significant overlap between patients with and without PE. The high positivity rates of the Geneva score and Wells criteria in both groups were unsurprising, as cases were selected from patients who performed CTPA. However, we might have expected cases with PE to have higher scores. The Wells score was marginally higher in patients with PE, and the Geneva score was similar between groups. The poor performance of the Wells criteria and the Geneva score might be attributed to the balanced distribution of key predictors across groups (e.g., malignancy, hemoptysis, tachycardia, recent fracture, and immobilization). In addition, both include physical signs of DVT, which might be difficult to assess in elderly, multimorbid patients.

Although our study design prevents us from making a decisive determination, the utility of current tools in the setting of pneumonia is questionable. Several small-scale studies have suggested that CXR infiltrates, history of chronic obstructive pulmonary disease or ischemic heart disease, abnormal body temperature, elevated CRP, and elevated troponin favor the diagnosis of pneumonia over PE [2, 3, 27, 30, 31]. In our study, CXR infiltrates were a prerequisite for inclusion and were therefore unhelpful for distinguishing between patients with and without PE. Other previously suggested factors were similar between the groups, with CRP being even higher in PE cases, although not sufficiently so to serve as a reliable diagnostic marker. This finding aligns with the previous studies demonstrating elevated inflammatory markers in this setting [2, 3]. Paparoupa et al. conducted a case–control study of 100 patients with pneumonia, with or without PE, but were unsuccessful in identifying a predictive model for an underlying PE [2]. Finally, we have found that the presence of HF was associated with a lower likelihood of detecting PE. This might reflect the contribution of HF to clinical features prompting CTPA in the first place, rather than a true reduction in PE risk.

Prolonged expiration or wheezing on auscultation were associated with a reduced risk for PE. Besides demonstrating the importance of physical examination, it also suggests that corresponding functional and sonographic findings might assist in this diagnostic challenge. Lung POCUS and echocardiography, both increasingly available in primary settings, should be tested for this indication [22, 23, 32]. We also showed that patients with PE had a higher rate of new-onset AF, a finding of interest from both mechanistic and diagnostic perspectives. However, this applies only to patients without prior AF, a common condition in the relevant age group [33]. New-onset AF has been reported in the setting of PE, mainly in those with heart failure (HF), ischemic heart disease, or massive PE [34]. Proposed mechanisms include right-ventricular stretch injury and the aggravation of comorbidities. Further directed research is necessary to assess the role of new-onset atrial fibrillation as a potential sign of hidden PE. Despite differences between groups, both abnormal auscultatory findings and new AF were present in only a small minority of patients, making their presence of limited diagnostic value. Finally, the incorporation of AI to aid in the initial assessment of possible PE cases across various clinical settings should be explored [24–26].

We found that 12.5% of patients with suspected pneumonia who underwent CTPA were diagnosed with PE. This significant CTPA positivity rate was higher than in some, although not all, previous reports in the general population, reaching as low as 5% [17, 35–38]. The rate remained significant after excluding subsegmental PE, with a 9.5% positivity rate for segmental or more proximal PE. Zhang et al. assessed the prevalence of PE in patients with community-acquired pneumonia [3]. However, CTPA was performed only in those with elevated D-dimer and clinical suspicion of PE, representing a high-risk subpopulation, and yielded 80 positive scans out of 139. Rohacek et al. reported that 9.7% of patients with signs of pneumonia on CTPA had concomitant PE [39]. Although the rate of co-existent PE and pneumonia varies based on the definition of pneumonia, the threshold for performing CTPA, and other factors, these studies, along with ours, highlight the significant prevalence of PE in patients with suspected pneumonia. Furthermore, future studies should address the potential underdiagnosis of PE in patients with pneumonia. It may be questioned whether CTPA should be prioritized over non-contrast CT to increase PE detection in patients with suspected pneumonia. However, our findings are currently insufficient to support a change in clinical practice. In an unselected population of pneumonia patients, the prevalence of PE may be lower than in our cohort, reducing the cost–benefit balance. Moreover, we did not observe an apparent impact of PE detection on clinical outcomes that would justify routine CTPA use.

No difference in mortality or other core outcomes was found between patients with and without PE (outcomes for subsegmental vs. larger PE are presented in Appendix A.3). However, these findings should be interpreted with caution. First, our study did not include all patients with suspected pneumonia and PE. Furthermore, virtually all diagnosed PE cases were treated with anticoagulation, and may have differed in other therapeutic measures from those without PE. Therefore, the true prognostic significance of PE in this setting remains uncertain.

Diagnosing PE has important long-term implications beyond its immediate therapeutic consequences, which could be overlooked if the diagnosis is missed. First, patients diagnosed with PE should be screened for underlying causes of hypercoagulability, such as malignancy, anti-phospholipid syndrome, and hereditary thrombophilia [1]. Additionally, timely diagnosis allows for appropriate follow-up and early detection of sequelae of PE, including CTEPH [1, 16, 17]. Early identification of CTEPH enables the early initiation of appropriate therapy, which has been shown to improve patient outcome [16, 17]. Moreover, up to 25% of patients with CTEPH have no known history of VTE [40]. These cases might be attributed to subclinical occurrences of acute VTE or to missed diagnoses in situations where current diagnostic tools fall short, such as in suspected pneumonia.

Our study has several limitations. First, it included only patients who underwent CTPA, thereby introducing potential selection bias into our cohort. As a result, patients with suspected pneumonia and a low presumed probability of PE were excluded. Conversely, as CXR before CTPA was a requirement, it is possible that patients with the highest likelihood of PE were omitted from this study. We thus were unable to evaluate the true incidence of PE in patients with suspected pneumonia. Furthermore, we might have underestimated the negative predictive value of current prediction scores when applied to the broader pneumonia population, which may have a lower PE incidence than our study cohort. A large proportion of reviewed cases were excluded, potentially contributing to selection bias. However, the majority of excluded cases had no infiltrate or no available CXR and were therefore not representative of the true pneumonia population. Furthermore, 12.6% of excluded cases had COVID-19, and their exclusion likely improved the generalizability of the results. The definition of pneumonia that was used is broad and debatable, although it aligns with previous studies [41, 42]. It could be argued that features included in the definition can be related to PE, including CXR infiltrates that may represent pulmonary infarction, atelectasis, or pulmonary edema. We therefore referred to the condition as ‘suspected pneumonia’. In addition, from a real-world physician's perspective, the possible diagnosis of an infectious process cannot be ruled out, given that an absolute diagnosis of a pulmonary infarct requires a biopsy and is, therefore, impractical [14]. As a result, pneumonia would have been suspected in many of these patients, influencing both evaluation and treatment decision-making. Third, the single-center study design limits the generalizability of our results. Nevertheless, our findings are based on a large patient cohort and rigorous inclusion criteria. Finally, specific populations were underrepresented, including patients with chronic kidney disease, in whom CTPA is less frequently employed.

In conclusion, PE is frequently observed in patients presenting with clinical signs of pneumonia. However, current diagnostic tools, such as D-dimer, CRP, and clinical decision tools, including the Geneva score and Wells criteria, may be inadequate for predicting PE in this population. Further prospective research in patients with suspected pneumonia is required to comprehensively characterize this entity, understand its implications, and develop improved diagnostic tools.

## Supplementary Information

Below is the link to the electronic supplementary material.

Supplementary file1 (DOCX 98 KB)

## Abbreviations

AF: Atrial fibrillation
AI: Artificial intelligence
AUC: Area under the curve
CAP: Community-acquired pneumonia
COVID-19: Coronavirus disease 2019
CTEPH: Chronic thromboembolic pulmonary hypertension
CTPA: Computer tomography pulmonary angiography
CXR: Chest ray
DVT: Deep vein thrombosis
ECG: Electrocardiogram
ICU: Intensive care unit
PE: Pulmonary embolism
POCUS: Point-of-care ultrasound
PPV: Positive predictive value
ROC: Receiver-operator characteristics
VIF: Variance inflation factor
VTE: Venous thromboembolism
