# Supplementary material for: Characterization of pulmonary embolism events in patients with suspected pneumonia
Source: Intern Emerg Med. 2026 Aug 18;21(8):2929–39. doi: 10.1007/s11739-026-04496-7 (PMC13751828; doi:10.1007/s11739-026-04496-7)
Supplement: Supplementary file 1 [file 11739_2026_4496_MOESM1_ESM.docx]

Characterization of Pulmonary Embolism Events in Patients with Suspected Pneumonia
Ariel Kenig ^1,2*^, Tal Sigawi ^1^, Josef-Daniel Shakargy ^1^, Fares Darawshy^2^, Rotem Kuint^2^, Henny Azmanov^2,^ and Neville Berkman^2^.

^1^ Department of Medicine, Hadassah Medical Center, and the Faculty of Medicine, Hebrew University, Jerusalem, Israel.

^2^ Institute of Pulmonary Medicine, Hadassah Medical Center, and the Faculty of Medicine, Hebrew University, Jerusalem, Israel

Journal: Internal and Emergency Medicine Journal

**Supplementary Material:**

ECG findings in patients with suspected pneumonia, with and without PE

Table S1 presents the prevalence of ECG findings in patients with and without PE. ECGs were obtained either from the original strips, which were analyzed by the researchers, or from clinician interpretations documented in the medical record.

**Table S1: ECG findings**

|  | No./N^a^ (%) | |  |
| --- | --- | --- | --- |
| **Characteristic** | **PE-negative**  N = 740 | **PE-positive**  N = 106 | **p-value** |
| Sinus rhythm | 577/658 (87.7) | 82/97 (84.5) | 0.38 |
| Atrial fibrillation or flutter | 71/658 (10.8) | 15/97 (15.5) | 0.18 |
| New-onset atrial fibrillation ^1^ | 20/71 (28.2) | 10/15 (66.7) | **0.004** |
| Incomplete RBBB | 55/656 (8.4) | 12/96 (12.5) | 0.19 |
| RBBB | 57/656 (8.7) | 9/96 (9.4) | 0.82 |
| Right axis deviation | 45/656 (6.9) | 9/95 (9.5) | 0.36 |
| S1Q3T3 | 27/656 (4.1) | 5/96 (5.2) | 0.59 |
| T wave inversions (leads 1-4) | 80/656 (12.2) | 11/96 (11.5) | 0.84 |
| T wave inversion (inferior leads) | 47/656 (7.2) | 6/96 (6.2) | 0.74 |
| ST elevation | 16/658 (2.4) | 3/96 (3.1) | 0.72 |
| ST depression | 54/658 (8.2) | 4/96 (4.2) | 0.17 |

^1^ New onset atrial fibrillation was defined as AF on admission without documentation of AF in the patient’s medical history. ECG, electrocardiography; PE, pulmonary embolism. RBBB, right bundle branch block.

Kaplan-Meier survival analysis

Figure S1 presents a Kaplan-Meier survival curve comparing patients with suspected pneumonia with and without PE. No significant difference in survival was observed between the two groups.

Figure S1: 90-day survival in patients with suspected pneumonia with and without PE. PE, pulmonary embolism.

Outcome according to PE type

PE cases were divided according to the involved artery into subsegmental PE and large PE (incorporating segmental and larger PE). Outcomes were compared between the two PE groups and patients with suspected pneumonia without PE.

**Table S3: Outcome of patients with subsegmental PE, larger PE, and those without PE**

|  | No. (%) | | |  |
| --- | --- | --- | --- | --- |
| **Characteristic** | **PE-negative**  N = 740 | **Subsegmental PE**  N = 20 | **Large PE ^1^**  N = 86 | **p-value** |
| Mortality |  |  |  |  |
| 30-day mortality | 87 (11.8) | 2 (10.0) | 6 (7.1) | 0.45 |
| 90-day mortality | 133 (17.9) | 4 (20.0) | 10 (11.6) | 0.30 |
| One-year mortality | 179 (24.1) | 8 (40.0) | 16 (18.6) | 0.13 |
| Hospitalization | 606 (81.9) | 17 (85.0) | 80 (93.0) | **0.023** |
| Length of stay, days (IQR) | 5 (2-9) | 6 (4-8) | 5 (3-13) | 0.26 |
| ICU | 58 (7.8) | 0 (0.0) | 12 (14.0) | 0.073 |
| Mechanical ventilation | 74 (10.0) | 1 (5.0) | 13 (15.1) | 0.75 |
| Vasopressors | 67 (9.1) | 1 (5.0) | 10 (11.6) | 0.67 |

^1^ Large PE was defined as any PE larger than subsegmental. ICU, intensive care unit; PE, pulmonary embolism.

Performance analysis of Wells and Geneva scores for detecting non-subsegmental PE.

Given the uncertain clinical significance of subsegmental PE, we evaluated the performance of the Wells’ criteria, Geneva score, and D-dimer testing in detecting segmental or more proximal PE among patients with suspected pneumonia who underwent CTPA.

**Table S4: Wells criteria and Geneva score for identifying non-subsegmental PE in suspected pneumonia**

A. Comparison of Wells criteria and Geneva score in patients with and without PE

| **Characteristic** | **PE-negative**  N = 470 | **PE-positive**  N = 106 | **p-value** |
| --- | --- | --- | --- |
| Wells score, mean (SD) | 1.70 (1.32) | 2.08 (1.59) | **0.048** |
| Geneva score, mean (SD) | 5.56 (2.32) | 5.71 (2.73) | 0.68 |
| Positive Wells criteria^1^, n (%) | 271 (98.2) | 49 (96.1) | 0.30 |
| Positive Geneva score^2^, n (%) | 221 (76.5) | 38 (69.1) | 0.25 |

B. Wells criteria, Geneva score, and D-dimer performances in suspected pneumonia

| **Test** | **Sensitivity** | **Specificity** | **PPV** | **NPV** |
| --- | --- | --- | --- | --- |
| Wells criteria (%) | 96.1 | 1.8 | 15.3 | 71.4 |
| Geneva score (%) | 69.1 | 23.5 | 14.7 | 80.0 |
| D-dimer > 0.5 mg/dL (%) | 96.0 | 2.0 | 14.3 | 75.0 |
| D-dimer > 1 mg/dL (%) | 94.0 | 14.3 | 15.7 | 93.3 |
| Age-adjusted D-dimer ^3^(%) | 96.0 | 5.1 | 14.7 | 88.2 |

^1^ Positive Wells criteria were defined as a score ≥2 or 0-1 combined with D-dimer levels > 0.5mg/dL. ^2^ A positive Geneva score was defined as a score of 4-10 with a positive D-dimer or a score >10 regardless of D-dimer levels. ^3^ Age-adjusted D-dimer cutoff relates to the increase of the upper normal limit (0.5mg/dL) by 0.1mg/dL for every decade above 50 years of age. NPV, negative predictive value; PE, pulmonary embolism; PPV, positive predictive value

Post hoc reduced multivariate analysis

A post hoc sensitivity analysis was conducted using a reduced multivariable regression model based on the main model, retaining age and additional variables from the original model that are directly relevant to the routine clinical evaluation of pulmonary embolism (signs of DVT, previous VTE, and D-dimer levels). Only D-dimer levels were independently associated with increased risk of PE in those with suspected pneumonia in the reduced model.

**Table S5: Reduced multivariate analyses of Parameters Associated with a Diagnosis of Pulmonary Embolism in Patients with Suspected Pneumonia**

| **Characteristic** | **OR** | **95% CI** | **p-value** |
| --- | --- | --- | --- |
| Age | 0.99 | 0.97 to 1.01 | 0.20 |
| Previous VTE | 1.64 | 0.73 to 3.69 | 0.23 |
| Signs of DVT | 1.79 | 0.66 to 4.86 | 0.25 |
| D-dimer, mg/dL | 1.02 | 1.00 to 1.04 | 0.015 |

DVT, deep vein thrombosis; VTE, venous thromboembolism.
